# What Role Does Substance Use Play in Intimate Partner Violence? A Narrative Analysis of In-Depth Interviews With Men in Substance Use Treatment and Their Current or Former Female Partner

**DOI:** 10.1177/0886260519879259

**Published:** 2019-10-03

**Authors:** Polly Radcliffe, David Gadd, Juliet Henderson, Beverly Love, Danielle Stephens-Lewis, Amy Johnson, Elizabeth Gilchrist, Gail Gilchrist

**Affiliations:** 1 King’s College London, UK; 2 The University of Manchester, UK; 3 University of Gloucestershire, Cheltenham, UK; 4 University of Worcester, UK; 5 The University of Melbourne, Victoria, Australia

**Keywords:** alcohol and drugs, domestic violence, vulnerability to abuse

## Abstract

Few studies have examined intimate partner violence (IPV) in relationships where one or both partners are in treatment for substance use, from the perspectives of both members of a couple. This study used thematic and narrative analysis of the accounts of 14 men recruited from substance use services and 14 women who were their current or former intimate partners. Separate researchers interviewed men and women from the same dyad pair. The psychopharmacological effects of substance use (including intoxication, craving, and withdrawal) were rarely the only explanation offered for IPV. Violence was reported to be primed and entangled with sexual jealousy, with perceptions of female impropriety and with women’s opposition to male authority. Both partners reported adversities and psychological vulnerabilities that they considered relevant to conflict and abuse. Male participants were more likely to describe IPV as uncharacteristic isolated events that arose from specific disputes—either aggravated by intoxication or withdrawal or about substance use and its resourcing—whereas women described enduring patterns of abusive behavior often linked to intoxication, craving, withdrawal, and to disputes linked to raising funds for substances. In relationships where both partners used substances, men described the need to protect their partners from addiction and from unscrupulous others while women described highly controlling behavior. In relationships where women were not dependent substance users, they reported the combined effects of psychological and financial abuse often linked to recurring patterns of substance use and relapse. These findings highlight the challenges faced by practitioners working with male perpetrators who use substances as well as the need of those working with women who have been abused to engage with the ways in which hesitance to leave male abusers can be complicated by shared drug dependency.

## Introduction

Historically, policymaking with respect to drugs, alcohol, and violence has focused on aggression in systems of drug distribution and supply (Executive Office of the President, 2016; Goldstein, 1985) and violence arising from intoxication in the night-time economy (Wickham, 2012). There is an increasing acknowledgment internationally, however, of the link between substance use and intimate partner violence (IPV) (Commonwealth of Australia, Department of Health, 2017; HM Government, 2017). The most recent U.K. Drug Strategy (HM Government, 2017) notes that women with experience of physical and sexual interpersonal violence are more likely to have drug or alcohol problems (Ellsberg et al., 2008) and that there is a higher prevalence of IPV perpetration among men in substance use treatment than in the general population (G. Gilchrist, Radcliffe, Noto, & Flavia, 2017; O’Farrell, Fals-Stewart, Murphy, & Murphy, 2003).

Although heterosexual men and people in same-sex relationships also experience IPV (Bailey, 2021; Kubicek, McNeeley, & Collins, 2016), the most common and severe forms of IPV are perpetrated against women by men (World Health Organization, 2013). Goldstein’s (1985) tripartite model of the relationship between general violence and drugs proposed that drugs and violence could be related in three ways: psychopharmacologically, economic-compulsively, or systemically. The psychopharmacological model emphasizes the direct effect of consuming or withdrawing from substances on violence perpetration; the economically compulsive model suggests that some drug users carry out violent crime to support their drug use; while the systemic model refers to “traditionally aggressive patterns of interaction within the system of drug distribution and use” (Goldstein, 1985, p. 497). Despite its failure to recognize either the interaction between social contexts and psychopharmacology (Parker & Auerhahn, 1998) or to consider the social and cultural contexts of substance-related offending more generally (Bennett & Holloway, 2009), Goldstein’s model has nevertheless been influential in shaping how the drug/violence “nexus” is conceived in government policy and in inspiring research on the disinhibitive effects of intoxication in specific social settings (Parker & Rebhun, 1995), including the family (Parker & Auerhahn, 1998).

Scholars of IPV have suggested that substance use may be the mechanism for reducing the threshold at which a perceived provocation would result in IPV for those who do not usually behave aggressively but not for those who are physically aggressive regardless of whether they are under the influence (Fals-Stewart, Leonard, & Birchler, 2005; Klostermann & Fals-Stewart, 2006). Self-report studies with perpetrators have found the strongest correlation between substance use and IPV perpetration among men who uphold values of male dominance (Field, Caetano, & Nelson, 2004; Peralta, Tuttle, & Steele, 2010). In a study that used a standardized scale to assess support for hostility toward women and male dominance, Renzetti, Lynch, and DeWall (2018) found that high levels of alcohol consumption have a greater impact on physical IPV perpetration for men identified as low in hostile sexism than for men high in hostile sexism. The authors conclude that there is a need for qualitative research to illuminate “how men’s constructions of normative masculinity and femininity contribute to levels of alcohol consumption and use of aggression against an intimate partner” (Renzetti et al., 2018, p. 203).

Other studies of domestic abuse have focused on IPV as gendered violence, conceptualized as part of a continuum within systems of patriarchal power (Kelly, 1998; Morgan & Thapar Björkert, 2006). Highly influential in the domestic violence service field, Pence and Paymar (1990) and Pence (1996) have argued that male “battering” includes “constellations of abuse” (Dobash & Dobash, 2004) that are intentional and tactical in character, deployed to ensure the exertion of power and control over female partners. Where substance use features in this scholarship, it is usually conceptualized as an attempt by men to excuse violence and abuse (Cavanagh, Dobash, Dobash, & Lewis, 2001; Galvani, 2004).

Conversely, a review of the psychiatric and psychological research over three decades suggest that the psychopharmacological effects of alcohol on violence and abuse should not be discounted and that “while neither a necessary nor a sufficient cause, excessive alcohol use does contribute to the occurrence of partner violence and that contribution is approximately equal to other contributing causes such as gender roles, anger and marital functioning” (Leonard & Quigley, 2017, p. 7). Moreover, Cafferky, Mendez, Jared, and Stith’s (2018) meta-analytic review reveals that substance abuse and dependence with related withdrawal and craving is more strongly associated with IPV perpetration than substance intoxication alone. They also highlight the need for qualitative research that is able to unpack the various contributions of intoxication and withdrawal/craving as perceived by perpetrators and survivors. A meta-ethnography of 26 such qualitative studies featuring separate IPV perpetrator and victim/survivor accounts in which one or both partners used substances, found both victims and perpetrators tended to link IPV perpetration to alcohol and stimulant drugs (methamphetamine and cocaine) intoxication (G. Gilchrist et al., 2019). In several studies, addiction and withdrawal were also found to make survivors vulnerable to IPV, particularly when both perpetrators and survivors were receiving treatment for, or were dependent on, substances (Macy, Renz, & Pelino, 2013; Watt, Guidera, Hobkirk, Skinner, & Meade, 2017). Perpetrators and survivors reported that irritability and agitation when “coming down” or “craving” alcohol (Satyanarayana, Hebbani, Hegde, Krishnan, & Srinivasan, 2015; Wilson, Graham, & Taft, 2017), heroin (Gilbert, El-Bassel, Rajah, Foleno, & Frye, 2001), methamphetamine (Abdul-Khabir, Hall, Swanson, & Shoptaw, 2014; Ludwig-Barron, Syvertsen, Lagare, Palinkas, & Stockman, 2015; Watt et al., 2017), and crack (Watt, 2012) resulted in violence among perpetrators (and sometimes survivors) who were dependent on substances. The partners of some substance using perpetrators also reported living in states of hypervigilance and suffering the overburden of managing households dominated by substance use and—nearly always in the context of scarce family resources and poverty—a range of forms of financial abuse. Across the 26 studies, IPV linked to substance use was played out in relation to unequal gender relations, in which male perpetrators sought to dominate and control their female partners. However, no studies in this review included accounts from both partners in abusive relationships. Moreover, few studies have examined IPV in relationships where one or both partners are in treatment for substance use, from the perspectives of both members of a couple. This limits our understanding of the relationship between IPV and substance use and of how we can effectively prevent and address such abuse. The current study addresses these gaps in understanding.

## Aims

In this article, we focus on how the themes of *Intoxication, Withdrawal and Craving*, and *Financial Abuse* featured in narratives about IPV perpetration and victimization. Our analysis seeks to furnish an understanding of the complex role of substance use, addiction, and substance using lifestyles in victimization and perpetration that can inform integrated interventions for men in treatment for substance use

## Methods

### Recruitment

Adult men were recruited from six community-based substance use treatment services in London and the West Midlands (England, United Kingdom) including the National Health Service (NHS) and voluntary sector services. Key workers at participating substance use treatment services were asked to identify male clients with a history of IPV perpetration. Prospective participants were approached by researchers in substance use treatment waiting rooms and given information about the study. Men were invited to take part in a short screening questionnaire prior to giving informed consent to check for eligibility to participate in a qualitative interview. The screening questionnaire focused on relationship status, substances used, and length of time in substance use treatment and included questions adapted from the WHO multi-country study on Women’s Health and Domestic Violence (Garcia-Moreno, Jansen, Ellsberg, Heise, & Watts, 2005) regarding whether prospective participants had ever been in domestically abusive relationships in which they or their partner would have encountered behavior consistent with psychological abuse, coercive control, financial abuse, and physical and sexual abuse. Inclusion criteria for the qualitative interviews included being 18 years or older, receiving treatment for alcohol or drug use from participating services, ability to be interviewed in English and answering positively to questions in the screening questionnaire about having ever perpetrated emotional, physical, and/or sexual abuse. Men subject to court orders preventing them from contacting their current or former partner were ineligible to participate.

A total of 37 male participants who took part were asked to provide contact details for their current or former female partner/s so that the research team could invite them to be interviewed. Of the 37, 27 men provided 32 contact details for their current or former partners. Three women declined to take part, 13 women proved non-contactable, and researchers were advised by staff not to contact two women who had recently relapsed drug use. In total, 14 current or former female partners agreed to take part in the study. This article focuses on the analysis of the interviews of these 14 heterosexual-couple dyads. Female participants were assured of the steps that would be taken to ensure their interview data would not be shared with their current or ex-partner. All participants were advised that there were limits to the confidentiality that could be afforded where unaddressed risks of harm and safeguarding issues to themselves or others were disclosed. Women and men in the same dyad were always interviewed by different researchers to ensure no information was inadvertently shared between participants, and the safeguarding protocol of the treatment service was followed to ensure the safety of women and their children. Both women and men taking part in the research were provided with contact details of support organizations for victim/survivors and perpetrators and paid £20 to compensate for their time.

Interviews with male and female participants mainly took place in counseling rooms in substance use treatment services. Where women were not willing or able to travel to substance use treatment services, interviews also took place in their homes or in a children’s center. One woman was interviewed via Skype as she had moved out of the area.

Interviews were conducted by five female interviewers, using an interview guide designed to elicit participants’ stories about substance use, relationships, and particular examples of abuse, using techniques adapted from the Free Association Narrative Interview Method (Hollway & Jefferson, 2008). The interview guide for men sought stories of substance use and perpetration, referring to men’s reports in the screening questionnaire of having perpetrated psychological, physical, sexual, and financial abuse. The interview guide for women asked them for stories about their relationships with male partners and former partners and about particular experiences of abuse. Interviews lasted between 37 and 96 minutes. Digital audio recordings of the interviews were anonymized at the point of transcription and checked twice for errors.

### Data Analysis

The analysis sought to integrate a thematic and narrative approach (Floersch, Longhofer, Kranke, & Townsend, 2010). A theoretically driven thematic analysis was conducted to identify the main ways in which substance use featured in male and female attributions and explanations for IPV (Braun & Clarke, 2006). Codes were derived both from the literature and our research question concerning how aspects of substance use explained IPV in both male and female dyad accounts and applied to a sample of transcripts by the first author and researchers, J.H., B.L., A.J. and D.S-L. using NVivo software for managing qualitative data. These codes included *Intoxication, Withdrawal and Craving*, and *Financial Abuse linked to Substance Use* (Table 1). Coding of all transcripts was then checked and refined by the first author in NVivo. Then the data were re-coded using strategies derived from narrative criminology that have analyzed violent offenders’ accounts (Brookman, 2014; Presser, 2004, 2009). At this stage, we examined the explanatory forms interview participants drew upon to account for their and their partners’ actions and in so doing, how they constructed their identities (Presser, 2004). Thus, we identified four overarching narratives through which male and female participants told their stories of IPV perpetration and victimization: *sexual betrayal/sexual jealousy, mutual combat/fighting back, protection/control*, and *psychological vulnerabilities*. This coding often exposed the contradictions between stated intentions and outcomes, as well as between the recollections of perpetrators and victims. Some men, for example, talked about using physical force to protect their partners, while women described the same behavior as controlling (Adams, Towns, & Gavey, 1995). As described by Brookman (2014), individual participants’ accounts often drew upon a range of attributions and narrative forms to explain IPV perpetration and victimization. For this reason, we do not attempt to quantify how many of the participants endorsed each theme presented.

**Table 1.** Themes and Narratives Description.

Theme	Description	
Intoxication	IPV, a result of intoxication from alcohol/crack/cocaine/stimulants	
Craving and withdrawal	IPV, a result of disputes linked to craving, coming down, and withdrawing from substances	
Financial abuse linked to substance use	Abuse linked to the need for money for the purchase of substance	
Narrative Explanation	Men’s Narratives	Women’s Narratives
Sexual betrayal/sexual jealousy	Male abuse arises from woman’s sexual betrayal or risk of betrayal	Male abuse arises from (often unfounded) sexual jealousy
Mutual combat/fighting back	Physical abuse in the context of a mutual “argument”	Female violence responding to male assault/attack
Protection/control	Male abuse linked to the need to protect partner from others	Male abuse associated with desire to control partner

*Note.* IPV = intimate partner violence.

While narrative theory typically explores the ways in which people’s recollections are temporally ordered (Presser, 2004, 2009), analysis of dyad interviews in which two participants recollect the same event differently complicates this task. Dyadic analysis benefits from identifying overlaps and contrasts in couples’ accounts (Eisikovits & Koren, 2010; Stern & Heise, 2018). In our integrated analysis, we counterpoised the explanations and attributions for IPV offered by both partners in the dyad, where possible, in relation to the same incidents. We compared dyad accounts, examining what pieces of evidence couples relied upon—particularly when it pertained to substance use—and what each individual party omitted from their accounts. We explored how, in explaining their own actions, participants depicted their own motives and characters, often in stark contrast to those they imputed to their current or former partners.

## Results

### Sample Characteristics

Male and female participants ranged in age from 28 to 56 (Table 2). Half the dyads continued to be in a relationship and half were separated. Relationships ranged in length between 3 and 26 years. Thirteen of the male partners and 12 of the female partners were white. Male participants, for the most part, used heroin and crack sometimes in combination with alcohol. All but two men were in treatment for heroin. They were often also continuing to use heroin, and/or crack cocaine, cannabis, and/or alcohol. The qualitative interviews revealed that six men and three women were housed in hostels or other temporary accommodation at the time of interview. One or both individuals in the dyad volunteered in the qualitative interviews that six of the females had never used heroin or crack, and only ever drunk alcohol “socially,” two of whom used cannabis. Four female participants reported currently using heroin and/or crack and four others had formerly used heroin and/or crack/cocaine (although in one case, her ex-partner claimed that she was continuing to use).

**Table 2.** Sample Characteristics.

Sample Characteristics		
	Men	Women
White	13	12
Nonwhite	1	2
Mean age	41 (*SD* 5.9)	41 (*SD* 9.7)^a^
Age range	33-50	28-56
Relationship Status		
Current relationship	7	7
Separated	7	7
Treatment for Heroin/crack	12	N/A
Treatment for Cocaine/alcohol	1	N/A
Treatment for Alcohol	1	N/A
Never used heroin/crack		6
Former user heroin/crack		4
Current user heroin/crack		4

a Women’s age values are based on the ages of nine of the women, as five women did not volunteer their ages.

Although we did not confirm reports of criminal justice involvement by consulting police records, eight men volunteered in the screening questionnaire that they had been arrested for IPV-related offenses and of these, six reported they had received custodial sentences. Eleven out of the 14 men described experiencing mental health problems, at least seven of whom had received a formal mental health diagnosis and psychiatric treatment. Two men described having experienced drug-induced psychosis. All eight women who were current or former users of heroin and or crack/cocaine described experiencing mental health problems and two women who were not heroin/crack users described the negative impact on their mental health of living in abusive relationships. Four women described having experienced IPV in previous relationships. Participants from three dyads revealed that children had been removed from their care or required by social services, to live with other family members.

In the qualitative interviews, many of the men described childhoods that were characterized by instability, including material deprivation, physical or sexual abuse, dropping out of school, and offending (Gadd et al., 2019). In addition, several men described having spent time in children’s homes or having lived with other family members. Men often reported witnessing domestic violence from their fathers to mothers from a young age, which they had found frightening and abhorrent. Men gave accounts of using alcohol, cannabis, and solvents in their teenage years and moving on to heroin and/or crack use in early adulthood. Predictably, male participants reported depressive symptoms, fighting with other men in the past year, experiencing a greater number of adverse childhood experiences and higher hazardous drinking scores (Breet, Seedat, & Kagee, 2019; Fulu et al., 2017; Gadd, 2002; G. Gilchrist et al., 2015; G. Gilchrist et al., 2017; Torrens, Gilchrist, Domingo-Salvany, & psyCoBarcelona Group, 2011).

### Intoxication

In the stories told by three separate dyads, intoxication from alcohol use on the part of the male partner was depicted as pivotal in disputes that escalated to violence but was rarely the sole explanation for it. Lisa, for example, described her mounting fury that her partner had been out all night, drinking with someone she described as a “first class prick” and a “wrong ‘un’”,

he got in with another prick . . . was out drinking every night, coming home at 5 o’clock and going to work at 6.30 drunk. And he . . . wouldn’t answer his phone . . . when I was ringing him I knew he was a wrong ‘un, he used to beat up on his girl, . . . but Ben wasn’t having none of it . . . and he came home one night really, really drunk and we got into an argument. He pointed in my face, so I bit him. (Lisa, treatment for heroin, Ben/Lisa dyad)

Lisa describes her response to Ben’s pointing in her face in a fight that had escalated from his late and drunken return to the house. Earlier in the interview, she had reported having experienced IPV in a previous relationship and had referred to the panic she had felt when Ben had “got right up in [her] face” and had explained “if you’re too close to us I panic, so I just lash out” (“us” in this context, refers to an idiomatic way of talking about the self, from the North East of England). Ben’s association with a “prick” who had “beaten up his girl” and his pointing in her face combined to explain her violent biting of his finger, in response to which,

He punched me in the face like you would hit a man. I was seeing stars and I felt my head pop. You could see the bone in my head, and I had two black eyes. I was like a panda. (Lisa, treatment for heroin, Ben/Lisa dyad)

Lisa could only explain Ben’s violent punch “like you would hit a man,” as a product of his intoxication.

Lisa: He’s not a violent person. He said, “No, no I’m sorry, I’m sorry, I didn’t [mean it].” It wasn’t Ben. It was the drink. I know that now it wasn’t him. It was the drink.

Interviewer: What do you mean by that?

Lisa: Because he’s not a violent person . . . I noticed how he changed when he was out drinking at night, I noticed the changes (Lisa, treatment for heroin, Ben/Lisa dyad)

As Ben remembered it, however, it was Lisa’s reaction—“going mad”—to him being “pissed” that caused the “argument” that precipitated his violence.

I remember being out with someone over there and her going mad that I was out with someone, for a long time and I come home pissed or drunk and yeah, that was—and then the argument kicked off and that was it. (Ben, treatment for heroin, Ben/Lisa dyad)

Ben said he deeply regretted punching Lisa, in part, because of the guilt it left him with but also because the need to make good had endured way beyond his initial apology:

I sort of froze as well . . . I was completely guilty for about—well, I still feel guilty now . . . I couldn’t believe it had happened and I, I felt like I was making it up to her for, for ages, you know. (Ben, treatment for heroin, Ben/Lisa dyad)

Both partners framed this incident as a single and aberrant act of violence that took place in the context of an otherwise loving relationship and that would not have occurred, but for alcohol intoxication. Although receiving treatment for heroin and crack use at the time of the interview, Ben reported that after this incident, he had barely drunk again.

For other dyads, such acts of violence had spelt the end of their relationships. Lucas had stayed behind at home and had “had a drink” while Bianca had taken the children out to a pub:

My wife was out with the four kids, from ten to eleven at night, and I’d had a drink, not a lot but I’d had a drink, and I was laid on the settee. They came in and my eldest son says to me, “We’ve been to a pub . . .” and I just (signalling that he flipped with his hands) . . . We had an argument, I didn’t slap her slap her, I just went (actions brushing her away), “Fuck off,” . . . and pushed her away . . . I’m going to bed.” So, I went in to get my baccy (tobacco) tin out, walked through—the rug was there. I tripped over the rug and fell on her, and I headbutted her. Then she told me to leave. So I left, but I was the one who phoned the police (Lucas, treatment for alcohol, Lucas/Bianca dyad)

While acknowledging that he had “had a drink,” Lucas suggested that the “argument” arose from his response to his wife having taken their children out “inappropriately” late in the evening to a pub. Lucas and Bianca placed differential emphasis on the role of intoxication in this argument and the violence that followed. Lucas minimized his violence, (“I didn’t slap her, slap her”), describing it as an accidental “trip” over the carpet as separate and unrelated to the slap that he presented as a justified rebuke, (despite reporting that he had called the police to admit some level of responsibility). Bianca meanwhile linked Lucas’s “shouting,” “name calling,” headbutting, and slapping of her to his inability to reason when “intoxicated”:

. . . you could tell he was intoxicated. He went to bed, he came back down 10 minutes later, started shouting again, name calling, carrying on, and I stood up to him and said, “Please go to bed, leave it and we’ll talk about it tomorrow.” He headbutted me and slapped me across the face. (Bianca, no drug use, Lucas/Bianca dyad)

In a third example of intoxicated violence, Thomas explained his alcohol and cannabis consumption as necessary to managing the pain of discovering his partner, Lucy, had been having an affair:

I found out that she’d been having an affair with him. I dealt with it the usual way, I did; drink, smoke. It was that night mainly where things came to a head. She slapped me, I punched her. There was a lot of screaming and shouting, shoving, pushing. (Thomas, alcohol and cannabis, Thomas/Lucy dyad)

Having described his use of substances as a way of coping with the distress of Lucy’s affair thus appealing to a discourse of sexual betrayal, Thomas made no link between his intoxication and the ensuing violence that he claimed was instigated by Lucy (“she slapped me”). His description of a mutual “fight” “a lot of screaming and shouting, shoving and pushing” provided what LeCouteur and Oxlad (2011) have described as “transactional warrant,” that is, justifying violence as part of a mutual exchange. This contrasted markedly with Lucy’s account, in which she depicted how Thomas had threatened to kill her in the presence of their children:

I remember him saying something about, “You and [him] together over my dead body.” He said, “I’ll kill you, and I’ll kill him,” and I took him dead serious. Why wouldn’t I, if someone says they’re going to kill me and they’re angry. At that point, he slapped me round the face . . . and I fell back on the sofa. (Lucy, no drug use, Thomas/Lucy dyad)

### Polydrug Intoxication, Betrayal, and Paranoia

While the events described by the three dyads above narrate apparently one-off acts of intoxicated violence that were responses to grievances that emerged in particular “arguments,” other participants described violence and abuse that endured over many years within which intoxication was entangled with men’s perceptions of entitlement and sexual betrayal. Jenny, who did not use drugs or alcohol, described a relationship lasting more than 20 years, in which her partner Mike was severely physically and psychologically abusive toward her. In his interview, Mike described use of cocaine, benzodiazepines, and alcohol and reported having been diagnosed with cocaine-induced psychosis. As well as perpetrating physical and psychological IPV toward Jenny, Mike also described having perpetrated severe violence against other men and having received a number of associated prison sentences. Jenny described—apparently random—acts of severe violence that often coincided with Mike’s intoxication with alcohol and cocaine, for example,

We was walking down the road. We’d come back from a club with my mum and he’d been drinking and taking drugs and I don’t remember why but . . . he grabbed me and strangled me, and a member of the public stopped him. (Jenny, no drug use, Mike/Jenny dyad)

Mike, by contrast, explained his need to physically police Jenny’s behavior, lest she be drinking with anyone who was not “family”:

when she goes out to the pub and that and she says she’s going with her family, I used to go there, see if there’s family there. When I see it’s not family, I’d go in the pub and drag her out of the pub, give her a smack, throw her in the fucking car and take her home, you know what I mean (Mike, treatment for cocaine and alcohol, Mike/Jenny dyad)

According to Jenny, Mike had been behaving that way since the birth of their first child:

just after I’d had the baby and, like it just started—we started up seeing each other again, that’s when he started getting controlling and it was like, well, I’m his Baby Mum. That’s what he used to say, so I had to do what he said. I had to go where he said or wherever he was. I mean we was always together, I weren’t allowed out by myself (Jenny, no drug use, Mike/Jenny dyad)

Other female partners gave accounts of how their partners’ jealous paranoia intensified when intoxicated. Rhian described Wayne’s behavior following his intoxication from alcohol and something he had “sniffed” as follows:

He came back, just drunk as anything, but I could tell he had had a sniff. His jaw was going, and his eyes were wired, and I could just tell. I was like, “You’ve done something . . . Who have you been texting?.” I said, “I haven’t been on my phone. I’ve literally been asleep.” He smashed my phone, because he was convinced that he had seen someone’s name on my phone that he actually hadn’t. (Rhian, no drug use, Wayne/Rhian dyad)

Wayne, by contrast, suggested conversely, that the problem was that he was insufficiently suspicious, or overly trusting of Rhian:

Some people, yes, it [cannabis use] might make them paranoid, but I’m not a paranoid guy. That’s why my girlfriend managed to cheat on me loads of times, because I’m not a paranoid guy. I’m not going to say to her, “No, you can’t go out,” or, “No, I’m going to follow you,” or, “I’m going to look at your phone.” (Wayne, treatment for heroin, Wayne/Rhian dyad)

By stressing that he was comparatively easy going by male standards, Wayne maintained it was his naïve lack of suspicion that enabled Rhian to cheat on him, thus also providing a moral assessment of her as deceitful. This invocation of the stereotype of the laid-back cannabis smoker appeared also to obscure a more general hostility toward and distrust of women.

Wayne: I thought in my mind that I felt like I was going to batter her a few times.

Interviewer: What happened before that for you to feel like it?

Wayne: . . . I had [a previous partner] cheating on me when I was younger, so I find it hard to trust girls anyway . . . but then Rhian knew all about it, and then just before Christmas the one year, she cheated on me, (Wayne, treatment for heroin, Wayne/Rhian dyad)

Rhian revealed that Wayne had done more than simply think about battering her:

I just remember him chasing me down to the end of the hallway, and then just pushing me over, and then when I was on the floor just kicking me and kicking me. (Rhian, no drug use, Wayne/Rhian dyad)

and that Wayne was very controlling, even timing her shopping trips and meetings with her mother:

He literally told me . . . “It will take you this long to walk there, this long in [name of supermarket], this long . . .” Do you know what I mean? (Rhian, no drug use, Wayne/Rhian dyad)

### Craving and Withdrawal

Other accounts of IPV centered on a need to raise money for drugs—amid craving and withdrawal—that was also construed through unequal gendered expectations. As previously described, four female participants in our study currently used, and three others had formerly used, heroin and or crack/cocaine. There were aspects of caring and compassion in some of these relationships (Simmons & Singer, 2006), with some dyads helping each other to avoid the symptoms of drug withdrawal. As Steve explained,

we both help each other get [drugs] . . . Like one day if I’ve got gear and she ain’t, she’ll come round, I’ll sort her out, and vice-versa. (Steve, treatment for heroin use, Steve/Loraine dyad)

Competition for drugs between partners and tensions surrounding how to raise funds to buy drugs to forestall craving and withdrawal were, nevertheless, common sources of disputes in relationships where both partners used drugs. Karen, and Tim, who used heroin, crack, and alcohol and who had been in a relationship for more than 20 years—interrupted by Tim’s several custodial sentences—described violence arising from a dispute over raising funds to buy drugs:

When we was both using, we used to shoplift, yeah, and, what happened, she said something to me and I said, I threw her on the floor, yeah, I think I was out of it, or something, but there was big hole, yeah, and she fell into the hole and the Police came . . . and I went to prison for it (Tim, treatment for heroin and crack, Tim/Karen dyad)

An argument about shoplifting to raise money for drugs provided Tim’s initial explanation for his violence: “she said something to me and I said, I threw her on the floor” before seeming to shift into an exculpatory reference to his intoxication, “I think I was out of it or something” to explain Karen’s “falling into a hole.” In contrast, Karen described a deliberate and brutal assault:

he’d beaten me up and pushed me down a manhole . . . and I was bleeding, my face was busted up . . . He had taken a big plank of, erm, rock . . . to chuck at me. I fell, he was fighting me, and I fell in the manhole and he was still hitting me with the stick. (Karen, heroin and crack use, Tim/Karen dyad)

From Karen’s perspective, Tim’s motivation for such violence was that she was not honoring their mutual obligations to provide for each other, typically by making money through illicit activity:

I went shoplifting. I made £100. I met my friend . . . stayed at [her] house for the day and I spent the money . . . He started hitting me and saying I’m taking him for a cunt and . . . he kicked out me out at 8 o’clock to make some money but I went shoplifting and I made some money, came back home. That was it, done for that day. (Karen, heroin and crack use, Tim/Karen dyad)

When Karen spent the money on her own drugs, she not only breached Tim’s expectation of sharing the proceeds of her shoplifting to source his own drugs but also challenged the authority he exercised. He responded by brutally kicking her out of the house and instructing her to make them both some money (see also Gilbert et al., 2001)

Joe and Kate, also both users of heroin and crack, described a dynamic in which disputes and violence revolved around how money was raised for drugs, when withdrawing or craving. Joe explained that withdrawal from heroin, led Kate, to fund her drug use by “clipping,” (stealing money from men she has agreed to have sex with):

if she’s sick, like she’ll go out clipping, yeah, . . . like robbing men; leading them on for sex but not giving it to them. Yeah, and I don’t like it, yeah. Right, it’s dangerous. She’s going to get herself hurt, yeah? I know what these blokes out here are like. They’re going to end up like bloody killing her or raping or something and that, yeah? So obviously that makes me, makes me and her argue. (Joe, treatment for heroin and crack use, Joe/ Kate dyad)

By contrast, Kate, who gave scant information about her drug use, leave alone how she funded it, described how early on in the relationship, Joe had shown love for her: “Yeah he was really nice, he was really nice at first. It was really cool like the way, he really spoilt me.” Subsequently, however, his attempts to control her had become stifling:

Like sometimes I feel like I can’t breathe, it’s like I can’t go out and he’s following me and he’s asking me where I am and . . . what I’m doing. (Kate, heroin and crack use, Joe/ Kate dyad)

Describing a specific incident where Joe’s controlling behavior tipped over into violence, Kate reported: “one time he got me on the floor he strangled me, he broke my ribs, he battered me,” providing a very different perspective on Joe’s claim that he protected her from harm. Joe also described violence perpetrated against him by Kate because she was craving drugs he would not pay for:

She hit me over the head with a hammer because I wouldn’t buy her drugs. Another time she put like a fireplace thing smashed me over the head and everything and that because she didn’t have no drugs and that. I’ve obviously done loads of things and everything and that. (Joe, treatment for heroin and crack use, Joe/Kate dyad)

Where both partners used substances, disputes also centered on men’s attempts to control with whom their partners used them. Often this was presented as a need to protect women from other unscrupulous men. David, for example, described slapping Julia in response to having found her in the act of using heroin with another man:

I saw red, I was so angry at the thought that she had got this other fella in the house, and he was smoking drugs in there . . . I caught the geezer giving her . . . heroin. I was just, I just saw red and I went mad. I wanted to get him out the house obviously and I was so angry with Julia for putting herself in that position and I did not help the situation by slapping her. (David, treatment for heroin, David/Julia dyad)

Meanwhile Julia—who divulged little in her interview about her own substance use—described the same incident, not as one single decisive event of David “seeing red” and removing her friend from the house, but as a sequence of events in which David had initially left her flat, subsequently returning to express his anger by attacking her, before starting to self-harm himself:

All of a sudden . . . the door just kicked in, and . . . he literally just pushed me and my head went against the toilet and then he went into the kitchen and got a knife and started cutting himself and then he went and my mate’s going, “I’m, I’m, I’m out of here.” (Julia, alcohol use, David/Julia dyad)

Access to both dyad accounts here revealed discrepancies in narratives of controlling versus protective behavior and the differential emphasis that perpetrators and victims give to the role of substance use in violent incidents.

### Financial Abuse

The dynamic of financial abuse was distinct in relationships where women did not use, or no longer used, substances. These dyad accounts were often highly discrepant, with women describing incidents that were wholly absent from men’s accounts. Lucy described discovering that her children’s father had sold their console games to buy drugs:

I was just like, no sign of a break-in. I knew their dad had been . . . They were absolutely heartbroken . . . The sun shone out of his backside . . . They’d saved money, they’d spent pocket money on them. It hurt. (Lucy, no drug use, Thomas/Lucy dyad)

For some women, demands for money were experienced as stressful and psychologically manipulative rather than physically threatening, for example,

Aggressive, no. But sometimes maybe [he] can use psychology for asking money. But not with the hands no. But always bothering me, stressing me. And this is not nice. But I don’t, I don’t want to see him when he’s [like] this. (Cheryl, cannabis use, Jason/Cheryl dyad)

Other women described how confrontations about relapsed drug use led to aggressive outbursts and physical violence. Gemma described how her partner Geoff’s persistent demands for money coincided with his clandestine relapse onto heroin use:

I just can’t understand it . . . It’s the devil and it just catches them. He keeps saying he’s beat it, he’s beat it, but it doesn’t seem like that because he’s lying to me all the time, and he’s having money off me all the time again. (Gemma, cannabis use, Geoff/Gemma dyad)

While Geoff denied any physical violence, minimizing his abuse as just “verbal”—“No, I’ve never abused her. Well, verbally abused her. It’s just that I’ve been a bit sharp”—Gemma described Geoff “flipping” when she asked him if he was using drugs again:

Because I approach him about the drugs. As soon as he knows I’m telling the truth I think that’s when he flips. He flips. It’s like he doesn’t like you to know. It feels like he likes to pull the wool over your eyes because he’ll say . . . He thinks I believe everything when I believe nothing now. (Gemma, cannabis use, Geoff/Gemma dyad)

Similarly, Mary described how exposing Matt’s relapsed drug use had provoked a physical attack:

while I’d been away [he] had started using proper back into the habit . . . And this day . . . I could feel the air change. He come into the front room and grabbed me round the throat and started saying that I was a busybody, getting up in his business. (Mary, former stimulant user, Matt/Mary dyad)

Matt, by contrast, said he was merely responding to Mary’s put downs—“abuse comes in all sorts of ways”—presenting himself as a victim of an apparently unreasonable woman, so insensitive to his own mental health problems that he had to cover her mouth to silence her:

I don’t think she gets the fact that abuse comes in all sorts of ways and also comes from when people are talking; putting you down constantly, constantly and screaming in your face. That’s abuse and so. Yeah, she wouldn’t shut up. That just cracks me up, you know, because I do have problem myself with mental health and everything. She just wouldn’t shut up and I put my hand over her mouth, you know; like pushed down on the settee and I put my hand over her mouth, screaming at her, “Shut up!” and yeah, eventually, she, she couldn’t breathe but I could have easily suffocated. (Matt, treatment for heroin and crack, Matt/Mary dyad)

Matt thus depicted his suffocation of Mary as a morally warrantable response to her criticism (see also Presser, 2004).

### Psychological Vulnerabilities

Most of the male and female participants in the study reported experiencing childhood and adult adversity and related mental health problems, including formal diagnoses (Table 1). Participants thus reported bringing acute psychological vulnerabilities to their relationships which were made relevant to their IPV through a range of narratives. For example, Mike suggested his own violence was a result of behavior that had become “normal” in his childhood, during which he saw his father abuse his mother:

I just didn’t like it ‘cause my dad was like always beating up my mum and stuff like that . . . So, so it’s normal to me, like. (Mike, treatment for cocaine and alcohol, Mike/Jenny dyad)

Likewise, Matt reported having been placed in local authority care as a young child, where he had been sexually abused. He had subsequently been returned to a children’s home after his grandmother, who had also cared for him, had had a stroke. Matt described how sexual abuse shaped his early drug use as well as his urge to be violent toward adult men as self-evident:

So obviously, when I got sent back into a children’s home when I was 13 it affected my mind so much that I just basically looked for things to stop thinking about stuff like that. You know, I was very violent, you know, growing up towards adult men. If they touched me, I just beat them up. (Matt, treatment for heroin and crack, Matt/Mary dyad)

In a more exculpatory narrative, Thomas stated that his ex-partner, Lucy, used her knowledge of his psychological vulnerabilities to provoke a physically violent “reaction” from him in arguments:

I didn’t really have much to do with my family because of my past drug use. They had washed their hands of me, so she’d [Lucy] constantly throw that in my face. My brother when he was in his late teens committed suicide. She’d throw that in my face as well, like, “Oh, is it any wonder your brother killed himself?” Doing her best to try and get a reaction from me. (Thomas, alcohol and cannabis, Thomas/Lucy dyad)

Other participants said that they used substances to cope with difficult feelings and to manage distress. Wayne described using heroin as a way of coping with feelings of loss and rejection:

Say if I see my daughter and she just walks on by, that’ll do me. It just does my head in, it makes me think all day, and then because I want to stop thinking about it, I go and take some drugs, but that doesn’t work. (Wayne, treatment for heroin and crack, Wayne/ Rhian dyad)

These accounts illustrate how psychological vulnerabilities feature in how participants rationalized their use of violence and how this violence tended to follow disturbing thoughts—sometimes evoked by justified criticisms from partners—that had long been suppressed through substance use.

## Discussion

While the psychopharmacological effects of substance use (including intoxication, craving, and withdrawal) featured in participants’ accounts of IPV, it was rarely the only explanation and appeared to be primed and entangled with narratives of sexual jealousy, male participants’ perception of female impropriety and women’s apparent opposition to male authority. Our analysis highlights, particularly for men who are poly substance users, an intimate playing out of “economic-compulsive” (Goldstein, 1985) abuse in disputes that frequently escalated from female partners’ attempts to oppose coercive control. In co-dependent, drug-using relationships, in particular, substance dependency and gendered power relations combined to make women vulnerable to abuse in disputes that centered on male partners’ control of drug supplies. Some male perpetrators also attempted to coerce women to raise funds to obtain substances and punished them physically when they failed to do so.

Our findings support those of Gilbert et al.’s (2001) that violence may be more likely where men are financially dependent on their partners. However exploitative, such relationships are not reducible to financial partnerships. Intimate relationships entail sexual vulnerabilities and emotional dependence requiring a trust that renders IPV perpetration, a source of shame for both perpetrators and survivors. The psychological vulnerabilities that both partners in our study frequently brought to these relationships made them ill equipped to negotiate the situational conflicts that arise from dependence on illicit substances in the context of scarce resources. The hostile sexism and general mistrust of women that were frequently evident in male participants’ explanations for perpetrating IPV, combined with their failure to fulfill the normative role of masculine provider moreover meant that their response to real—and imagined—sexual betrayal was frequently both a means of reasserting patriarchal authority and a denial of shame.

In their study of 95 couples where men had been arrested for domestic violence–related offenses, Dobash and Dobash (2004) found men and women disagreed about the nature, frequency, and impact of men’s violence. Likewise, Hydén (1994) found in her study of 20 Swedish couples that men were more likely to depict violence as bilateral and transactional elements of disputes and arguments that had escalated, while their female partners referred to the violence as assaults. Our study elaborates the differential narratives through which such gender differences are played out where one or both partners use and are in treatment for substance use. Male participants described intoxication with alcohol and cannabis and craving and withdrawal from heroin and crack leading to isolated incidents of perceived uncharacteristic violence in the context of escalating disputes. They commonly justified such loss of control as a result of female impropriety, sexual jealousy, and betrayal, the latter of which could include criticizing the men for their failings and drug use. For female partners, by contrast, such violent incidents were more likely to be described in the context of patterns of abusive behavior, at the extreme end of which included paranoid, highly coercive control, and brutal violence. Women described experiencing threats and physical abuse as punishment for disagreeing with or challenging their partners’ authority and control. While men were more likely to describe their violence as transactionally warranted, women described attacks initiated by male partners who judged them for not making sufficient effort to raise funds and hence defaulting on an unspoken commitment to share money and supplies that were often earned illicitly.

Where both partners used multiple substances, men described using violence and control to protect their partners from “addiction” and from unscrupulous others while women described having to “earn” access to substances, restrictions on who they could use substances with, and using violence themselves to resist male control. In relationships where women were not dependent substance users, economic abuse on the part of male partners was also associated with women’s resistance to male perpetrators attempts to take funds from them and sell their belongings to fund secret use. Women frequently described these experiences additionally as confusing and psychologically abusive since the rationale for the men’s violence was partially obscured.

Access to the narratives of both partners in abusive relationships provides insights into the dynamic of IPV perpetration by men in treatment for substance use. It is in highlighting the differences between how men and women in the same couples tell these stories that our study is unique (Neal & Edwards, 2017) and has the potential to inform treatment. Longitudinal qualitative dyad studies are needed to understand how IPV and substance use impact relationships and substance use over time. A core question these findings raise for treatment practitioners is how men who have perpetrated abuse can be helped to recognize that what they see as isolated incidents in which substance use occasionally causes things to get out of control, is part of a different more troubling story from the perspective of women who feel intimidated, dependent, and ashamed. This is a particular challenge in practice where the actual partners are not brought into the room with perpetrators but research and structures of accountability with women’s services can fill this gap. Our findings support the need for interventions for such men that concurrently address the complex interconnections of IPV with substance use (E. Gilchrist et al., 2003; G. Gilchrist & Hegarty, 2017) and services for women that are informed by an understanding of how dependence and withdrawal frame disputes. Our study suggests specific ways in which men’s narratives that rationalize IPV can be reframed and through which tendencies to control and dominate their female partners can be challenged and behavior changed. As we have shown, some men’s desire to protect their female partners from substance use or predatory substance users was, from these women’s perspectives, primed by intoxication, sexual jealousy, and controlling tendencies. Efforts at this controlling protectionism could lead to what the men regarded as accidental violence or outbursts that were, in hindsight, excessive but otherwise regrettable and out of character. Some women omitted accounts of their own violence and substance use from recollections of incidents that led to them being severely assaulted in ways that sounded much more callous and deliberate than their partner’s recognized or were prepared to admit. There is thus a need to provide support to women who are subject to IPV—some of it being life threatening—but who fall short of “ideal” notions of “victimhood” and whose lives are also complicated by adverse childhood experiences, mental health problems, and substance use in much the same ways as their abusers’ lives have been. Trials of integrated interventions for men and women who use substances and perpetrate or experience IPV are needed to test their effectiveness in improving relationships, reducing IPV, and substance use.

## Strengths and Weaknesses

A strength of this study was the opportunity it afforded to analyze the accounts of partners in the same relationship, enabling us to compare and contrast men and women’s perspectives and representations of IPV and substance use. Competing dyad accounts complicated analysis of the temporal order of events, substance use and IPV, meaning it was not always clear whether substance use preceded the IPV or vice versa. The social desirability and underreporting that are frequently a limitation in IPV research are thus also a feature of our analysis. The fact that we had a community rather than criminal justice sample made possible the analysis of accounts of everyday and sometimes mutual IPV perpetration that may be more characteristic of the substance using population. Our three nonwhite participants were all recruited from services in a London borough, reflecting its treatment population as a whole (personal communication, R. Gray) and which, as a proportion of the 28 participants in both sites, reflects the ethnic makeup of the national treatment population (Public Health England, 2018). We have described the protocol followed in terms of disclosure and limits to confidentiality. Although we are aware that this may have limited participants’ openness, we believe our data are nonetheless rich in disclosures and conceptually illuminating.
